# Assessment of tobacco imagery and compliance with tobacco-free rules in popular Indian films

**DOI:** 10.1136/tobaccocontrol-2018-054613

**Published:** 2019-02-16

**Authors:** Muralidhar M Kulkarni, Veena Ganesh Kamath, Jo Cranwell, John Britton, Gaurang P Nazar, Monika Arora, Kirthinath Ballal, Asha Kamath

**Affiliations:** 1 Community Medicine, Kasturba Medical College, Manipal Academy of Higher Education, Udupi, Karnataka, India; 2 Tobacco Control Research Group, Department for Health, University of Bath, Bath, UK; 3 UK Centre for Tobacco and Alcohol Studies, Division of Epidemiology and Public Health, University of Nottingham, Nottingham, UK; 4 HRIDAY, Delhi, India; 5 Health Promotion and Tobacco Control, Public Health Foundation of India, Delhi, India; 6 Department of Statistics, Prasanna School of Public Health, Manipal Academy of Higher Education, Udupi, Karnataka, India

**Keywords:** media, low/middle income country, public policy

## Abstract

**Background:**

Exposure to smoking in films causes smoking uptake among adolescents. Investigation of the extent to which tobacco imagery appears, or tobacco control laws are complied with in Indian films is limited, and especially so for films in regional languages. This study presents an analysis of tobacco content and compliance with tobacco control laws in popular films in several languages from the Karnataka state of India.

**Methods:**

We used 5 min interval coding to measure actual tobacco use, implied tobacco use, tobacco paraphernalia and tobacco branding in the top 10 films identified from national box office ratings and regional distributor reports in Karnataka in 2015 and 2016. We also assessed compliance with tobacco-free film rules in India.

**Findings:**

A total of 47 films, in English, Hindi, Malayalam, Tamil, Telugu and Tulu languages were coded. Any tobacco imagery was observed in 72% of films, and actual tobacco use in 50%. Tobacco imagery was equally prevalent in films classified as suitable for universal viewing (U category) or at age 12 or more (U/A category) films; and significantly more common in films made in regional than national language (Hindi). None of the films were fully compliant with legal requirements on health spots, audiovisual disclaimers and health warnings.

**Conclusions:**

Tobacco content was common in films classified as suitable for viewing by children, more among regional than national languages. Compliance with tobacco control laws was low. Stricter enforcement of tobacco-free film rules will protect children and adolescents from exposure to tobacco use on screen.

## Introduction

Tobacco smoking currently kills more than 7 million people a year^1^ and addiction to smoking is typically established during teenage years.^2 3^ There is growing evidence that uptake of smoking among young people is caused, in part, by exposure to tobacco imagery in films.^4–6^

India is a leader in film production, generating more than a fifth of the global total of films produced each year, and including films in national (Hindi) and a range of regional languages.^7^ Tobacco-free film rules implemented in India in 2003 under Section 5 of Cigarettes and Other Tobacco Products Act (COTPA) and amended last in 2012 prohibit paid tobacco product placement in films, require all films containing tobacco imagery to include a 20 s audiovisual health warning at the beginning and in the middle of the film, and require visual health warnings to be displayed on the screen when tobacco products or use are shown.^8 9^ However, studies of tobacco imagery are limited to films in national (Hindi) language,^10 11^ leaving regional language films relatively unexplored. This study aims to quantify tobacco imagery and evaluate COTPA compliance in the most popular films in the Karnataka region in Southern India, including those made in regional languages such as Kannada, Tulu, Telugu, Tamil and Malayalam.

## Methods

### Selection of films

We used national box office ratings^12^ to identify the 10 highest revenue national films, and asked each of the two leading film distributors in the Udupi district of Karnataka to identify the 10 highest revenue films, for both 2015 and 2016. After removing duplicates, this process identified 47 films to be coded for tobacco content.

### Coding method

Movies were coded in 5 min intervals using previously described methods,^13^ recording the presence or absence in each interval of:

Actual use: Use of tobacco onscreen by any character.

Implied use: Implied but not actual use on screen, verbal or non-verbal.

Tobacco paraphernalia: Presence of tobacco products or related materials such as matchboxes, lighters, ashtrays and smoking signage.

Brand appearance: Presence of clear, unambiguous, tobacco branding.

Any tobacco content: Any of the above.

An occurrence in any of the above categories was coded as present or absent in each interval; multiple appearances in the same category in the same interval were coded as a single event, while the same appearance transitioning into two or more intervals was coded as a multiple event. Brand appearances were recorded as the total number, by brand name, in each interval. The tobacco products coded included cigarettes, bidis, cigar, pipe and chewable tobacco. We also looked for electronic cigarette use.

For each film, we determined whether health spots, audiovisual disclaimers and static warning messages were included. Static messages were observed for legibility and whether they were placed prominently at the bottom of the screen, in black font on white background, and used the messages ‘smoking causes cancer’ or *‘*smoking kills*’* for smoked tobacco, or ‘tobacco causes cancer’ or ‘tobacco kills’ for chewing and other smokeless forms of tobacco in the language of the film.^9^ Two researchers independently coded the films, and coding differences resolved by discussion with a third researcher.

### Data analysis

Data were entered into Microsoft Office Excel 2016 and analysed using Excel and STATA V.15 software using simple descriptive statistics. Content was compared between films in the different Central Board of Film Certification (CBFC) classification categories (U- unrestricted public exhibition; UA–unrestricted public exhibition, but with a word of caution that parental discretion required for children below 12 years and A-restricted to adults)^14^ using the χ^2^ test.

## Results

We coded 11 Hindi, 5 Kannada, 3 English and 6 other language (Tulu, Tamil, Telugu and Malayalam) films from 2015, and 8 Hindi, 6 Kannada, 4 English and 4 other language films from 2016. Among the 47 films, 19 (40%) were U; 26 (56%) UA and 2 (4%) were A rated by CBFC. The coded films ranged from 101 to 176 min in length (mean (SD) run time 146.2 (15.5) minutes) and included a total of 7029 min which we coded in 1396 5 min intervals.

### Tobacco content in all films

#### Any tobacco content

Any tobacco content occurred in 14% of the total intervals in 34 (72.3%) films with equal prevalence in U (85 intervals containing any tobacco in a total of 576 intervals, 15%) and UA classified (110 out of 764 intervals, 14%) films (table 1).

**Table 1:** Tobacco content as per CBFC age rating and language of the films

Tobacco content and CBFC age rating:				
CBFC age rating	Total (%) intervals			
	U	UA	A	Total
All intervals	576 (41)	764 (55)	56 (4)	1396 (100.0)
Any tobacco	85 (43)	110 (56)	2 (1)	197 (100.0)
Actual tobacco use	54 (39)	83 (60)	1 (0.7)	138 (100.0)
Implied tobacco use	13 (48)	13 (48)	1 (4)	27 (100.0)
Tobacco paraphernalia	18 (56)	14 (44)	0	32 (100.0)
Tobacco branding	2 (50)	2 (50)	0	4 (100.0)

Tobacco content in films by language of film				
Language	English	Hindi	Regional languages	Total
Any tobacco content	2 (6)	13 (38)	19 (56)	34 (100.0)
Actual tobacco use	1 (4)	7 (29)	16 (68)	24 (100.0)
Implied tobacco use	1 (6)	7 (39)	10 (56)	18 (100.0)
Tobacco paraphernalia	1 (5)	6 (29)	14 (67)	21 (100.0)
Tobacco branding	0	1 (25%)	3 (75%)	2 (100.0)

#### Actual tobacco use

Actual tobacco use appeared in 24 (51%) films of which 12 (50%) were U rated, 11 (45.8%) UA rated and 1 (4%) was A rated. Actual use involved cigarette smoking in 34%, cigar in 4.3%, pipe smoking in 2% and mixed tobacco product use in 11% of films. Mixed tobacco use was shown in 3 (16%) of U-rated and 2 (8%) of UA-rated films.

#### Implied tobacco use

Implied tobacco use appeared in 18 (38%) films with 1 (6%), 8 (44%) and 9 (50%) being A, U and UA-rated films, respectively. Non-verbal implied use (eg, a lit cigarette placed on a table) occurred in 12 (26%) and verbal implied use (eg, an actor saying that he had not used a cigarette for many days) in 4% of the films.

#### Tobacco paraphernalia

Tobacco paraphernalia appeared in 21 (45%) films including 10 (48%) in U and 11 (52%) in UA films. The most common paraphernalia appearances involved cigarettes (7/21), matches/lighters (7/21), smoking signage (1/21) and ashtray (1/21). Five films had a combination of paraphernalia types.

#### Tobacco branding

Brand appearances occurred in nine (1%) intervals and four films, two being U rated and two UA rated. The brands comprised Passing Show cigarettes and a range of smokeless brands (Madhu, Vimal, Chaini, Siddu and Maruti).

#### Electronic cigarettes

There were no electronic cigarette appearances in any of the coded films.

### Tobacco content by language of film

#### Actual tobacco use

Different language films differed in the occurrence of imagery of actual tobacco use with occurrences in 25% (1/4) English, 37% (7/19) of Hindi and 76% (16/21) regional language films. Regional language films had significantly higher (p<0.05) occurrence of any tobacco content, actual use and tobacco paraphernalia than Hindi and English films (table 1).

### COTPA adherence

Among the 34 films containing any tobacco imagery, health spots at the start and middle of the films appeared, respectively, in 10 (29%) and 5 (15%) films. Only eight films (24%) included an audiovisual disclaimer at the beginning and none in the middle of the films. Of the 165 intervals identified containing actual or implied tobacco use, 144 (87%) displayed static messages of which 132 (92%) were non-compliant due to non-inclusion of the approved text, not being in the language of the film or poor legibility, or not in the specified format of a black font on white background. Adherence to regulations was generally higher in films made in local languages than in Hindi or in English, but was in all cases low.

## Discussion

This study demonstrates that tobacco content is common in popular Indian films, and particularly in regional language films, regardless of age classification; and that compliance with tobacco-free film rules is low. Our study also demonstrates tobacco content in a higher proportion of films than in previous studies from India and elsewhere.^11 13 15^ The particularly high occurrence of tobacco imagery in regional language films suggests that these films may represent a significant source of exposure in Indian children.

Coding films is time-consuming, so we limited our study to a sample of nationally and locally top-grossing films as these are likely to have the highest audiences. We used 5 min interval coding as a logistically feasible, semiquantitative measure of film content based on methods used successfully in a range of other studies^13 16^ and thus providing results directly comparable with work in other countries. Our study was limited to only one region of India, so further work is required to determine whether our findings reflect the content of films from other areas, which include 29 states and films in 35 different languages.^17^

The importance of preventing exposure of adolescents to tobacco imagery in films is recognised by WHO’s Framework Convention on Tobacco Control, which recommends adult ratings for films depicting tobacco imagery.^18^ In India, the original COTPA legislated for this but was overturned through a legal appeal, leading to the introduction of the 2012 requirements for health spots, audiovisual disclaimers and health warning subtitles.^9^ India is, however, implementing several measures to combat tobacco use, and has recorded a reduction in the prevalence of tobacco use in recent years. This is also true of Karnataka, where the prevalence of tobacco use among adults and youth, respectively, fell from 28.2% and 6.8% to 22.8% and 3.6% in the 7 years between the Global Adult Tobacco Survey (GATS) I and II surveys conducted in India.^19^ Our findings identify a need to step up the efforts to further reduce the tobacco burden by stronger measures to reduce exposure of young people to tobacco imagery in films.

**What this paper adds**

- Tobacco imagery in Bollywood films has been shown to generate high levels of large population-level exposure in India.
- This article is the first to explore tobacco imagery and assess compliance with current Indian movie-related tobacco control laws in films produced in regional languages, commonly used in Karnataka, India.
- The study shows that regional language films contain higher levels of tobacco imagery than those made in national (Hindi) or English language.
- The study also shows that full compliance with legal requirements to include antitobacco health spots, audio–video disclaimers and on-screen health warnings against tobacco imagery is low in films popular in Karnataka, India.
